# Different Placement Practices for Different Families? Children’s Adjustment in LGH Adoptive Families

**DOI:** 10.3389/fpsyg.2021.649853

**Published:** 2021-06-18

**Authors:** Pedro Alexandre Costa, Fiona Tasker, Isabel Pereira Leal

**Affiliations:** ^1^University Institute of Psychological, Social and Life Sciences (ISPA), Lisbon, Portugal; ^2^William James Center for Research, Lisbon, Portugal; ^3^Department of Psychological Sciences, Birkbeck, University of London, London, United Kingdom

**Keywords:** adoption processes, early adversity, resilience in adoption, lesbian mothers, gay fathers

## Abstract

**Objective:**

The purpose of this study was to examine the characteristics of children placed with lesbian, gay, and heterosexual adopters, and to examine children’s problem behaviors and positive psychosocial adjustment across the three family types.

**Background:**

There is evidence that children with hard-to-place profiles may be more likely to be matched with lesbian and gay parents. In addition, children adopted from care face greater developmental difficulties than children raised by their birth families, although adoptive parents may buffer the negative effects of early adversity on their children’s psychosocial adjustment.

**Method:**

A final sample of 149 adoptive families from across the United Kingdom was recruited: 71 heterosexual parented, 39 lesbian parented, and 39 gay parented.

**Results:**

The results showed that gay and lesbian parents were more likely than heterosexual parents to be matched with hard-to-place children, partially because they were more open to being matched with children with hard-to-place profiles. However, no differences among the three family types on children’s psychosocial adjustment were found, when controlling for children’s early adversity.

**Conclusion:**

Adopted children displayed similar levels of problem behaviors and positive adjustment in lesbian, gay, and heterosexual parented families. Early adversity and having a physical problem/disability accounted for much of the variance in problem behaviors whereas parenting did not. In contrast, it was suggested that parenting processes, namely, parental closeness, may help to explain children’s positive adjustment.

## Introduction

There is some evidence that lesbian and gay parents disproportionally adopt children with different arrays of difficulties – or *hard-to-place* children – due to discriminatory practices in the adoption process (Brooks and Goldberg, 2001; Brodzinsky, 2011). However, little is known about whether this is attributable to open discriminatory practices, implicit biases within the adoption system, or differential placement are based upon lesbian and gay parents’ greater openness to adopt hard-to-place children. If lesbian and gay parents are being differentially treated in the adoption assessment process, and matched with hard-to-place children, this may in turn stack the odds against a successful adoption.

Research concerning children’s psychosocial adjustment in adoptive families thus far has focused mostly on the prevalence of child problem behaviors. While there is evidence that children adopted from public care may present higher rates of problem behaviors than children living with their birth families (Miller et al., 2000; Howard et al., 2004; Grotevant and McDermott, 2014), adoption is advantageous in promoting child positive adjustment and general well-being when compared to alternatives such as fostering or institutional care (e.g., Triseliotis, 2002; Juffer and Van IJzendoorn, 2009). Specific to adoptive lesbian and gay parented families, greater attention to family processes within these families and its effects on children’s psychosocial adjustment and well-being are needed (Farr et al., 2020), as these may differ from those in heterosexual parented families due to, for example, differential placement practices. For example, in the United States. Farr et al. (2010) reported that adopted children’s internalizing and externalizing problems did not differ as a function of parents’ sexual orientation. In the United Kingdom, Golombok et al. (2014) found that adopted children with lesbian or gay parents scored significantly lower on externalizing problems and similarly on internalizing problems when compared to adopted children with heterosexual parents, but few differences between the families were found when the children reached adolescence (McConnachie et al., 2021).

The purpose of this study was to (1) examine whether lesbian and gay parents were more likely than heterosexual parents to adopt hard-to-place children, and if any differences in placement were associated with parents’ own preferences, and (2) examine adopted children’s profiles and its association with problem behaviors (internalizing and externalizing problems, and negative affect) and positive psychosocial adjustment (prosocial behaviors, and positive affect).

### Psychosocial Adjustment of Children Raised by Lesbian, Gay, and Heterosexual Parents

After over 40 years of research into lesbian and gay parented families, a scientific consensus has been achieved in that (1) lesbian women and gay men are at least as capable as heterosexual women and men of raising well-adjusted children, and (2) lesbian and gay parents’ sexual orientation does not negatively impact their children’s psychosocial adjustment (Crowl et al., 2008; Fedewa et al., 2015; Carneiro et al., 2017). Review studies have identified three main waves of research into lesbian and gay parented families (Golombok, 2007; Johnson, 2012). The first wave of research was focused on lesbian, gay, and bisexual parents who became parents within heterosexual relationships before disclosing their sexual identity, and who were mostly compared to heterosexual parents and their children as a norm group. Some research about these family trajectories continues (for a recent review, see Tasker and Lavender-Stott, 2020). Nevertheless, by the mid to late-1990s’ there was a shift toward studying mostly lesbian parented families with children conceived through Assisted Reproductive Technologies (ART) or self-insemination with a sperm donor. The first longitudinal studies in the United States (Gartrell et al., 1996) and later in the Netherlands (Bos et al., 2004) were conducted to investigate the effects of having two same-gender parents on family dynamics and children’s psychosocial adjustment. This second wave of research was made possible by both growing social acceptance and legal recognition of lesbian and gay parented families across the western world and reflected a phenomenon now known as the *lesbian baby boom* (Patterson, 1992). More recently, the third wave of research has started to question the heteronormative perspective of previous research that has used heterosexual parented families as a norm to conform to and has urged academics to look beyond simple comparisons and examine the specificities of LGBT + parented families and their different family trajectories (Stacey and Biblarz, 2001).

Despite considerable diversity in the family trajectories of LGBT + parents, the study of lesbian mothers with children conceived through ART and donor insemination have now dominated the field and few studies have examined the experiences of gay and bisexual fathers, or of gender non-conforming parents (e.g., Carneiro et al., 2017). Research have only recently started to examine the experiences of gay fathers who had children through surrogacy, both in and outside the US (e.g., Bergman et al., 2010; Golombok et al., 2018; Shenkman et al., 2020). Pockets of research on adoption by lesbian and parents have been identified (for a recent review, see Farr et al., 2020).

It has been reported that same-gender couples are more likely than different-gender couples to choose adoption as their preferred route to becoming parents and that they are more likely to effectively adopt children at least in the United States (Mallon, 2011; Goldberg and Conron, 2018). However, there is also an additional difference between lesbian and gay adopters in that lesbian mothers were more likely than gay fathers to have considered and sought biological parenthood before arriving at the decision to adopt (Mellish et al., 2013; Costa and Tasker, 2018). Most research assessing lesbian and gay adoptive parents and their children has found few differences when compared to heterosexual adoptive parents and their children, and cumulative evidence attests that children with lesbian and gay parents do not show higher levels of behavior problems, gender atypicality, or difficulties in their overall psychosocial adjustment compared with those with heterosexual parents (for a comprehensive review, see Farr et al., 2020). Nevertheless, most of this research has been conducted in the United States, often within the private adoption system. A few studies from other parts of the world, namely, from Europe, have reported the challenges and difficulties prospective LGBT + parents face in navigating adoption systems which are dominated by welfare adoption orders (Messina and D’Amore, 2018).

### Assessment of Lesbian and Gay Adopters and Placement of Children

There is evidence that lesbian and gay prospective adopters may encounter different levels of discrimination within the adoption system. Early studies from the United States have shown that lesbian and gay applicants faced greater scrutiny than heterosexual applicants regarding their parenting ability and their gendered parenting roles, suggesting a heteronormative view of family (e.g., Ryan, 2000; Brooks and Goldberg, 2001). Whereas some lesbian and gay single applicants and same-gender couples experienced discrimination or were denied the possibility of adoption by the adoption agency or State laws, others faced covert discriminatory practices by finding themselves matched only with hard-to-place children (Brodzinsky et al., 2002). Similarly, early United Kingdom studies have reported that lesbian and gay applicants were perceived as “the last resort” for children who would not be adopted by heterosexual couples (e.g., Hicks, 1996).

Mounting evidence has indicated that lesbian and gay adopters are more likely to be placed with children with hard-to-place profiles (Brodzinsky, 2011; Lavner et al., 2012; Golombok et al., 2014; Costa and Tasker, 2018). Children who have experienced pre-adoption trauma and adversity may consequently develop physical or health problems and may also be at higher risk for the development of psychological, cognitive, and learning disabilities, which in turn negatively affect both their behavioral and emotional adjustment and overall family functioning (Rutter, 2000; Erich and Leung, 2002; Nalavany et al., 2008). In the United States, Lavner et al. (2012, 2014) reported that young children (up to 24 months of age) placed with lesbian and gay parents had significantly more pre-adoption risk factors than had children placed with heterosexual parented families, although these factors did not significantly affect either child or parent adjustment. Another United States based study (Averett et al., 2009), with a large cohort of lesbian, gay, and heterosexual parents who had adopted children aged between 1.5 and 18 years found contrasting results; The authors reported that heterosexual parents were more likely than lesbian and gay parents to have adopted children with preadoptive experiences of trauma, namely neglect and physical abuse. Nonetheless, no differences on children’s psychosocial adjustment across family types were found, and the authors concluded that “the sexual orientation of the adoptive parents in this sample had no significant impact on the internalizing or externalizing behaviors of the children” (Averett et al., 2009, p. 143).

Other studies have reported that gay parents were more likely to be matched with older children and with children who had spent longer periods of time in care (Mellish et al., 2013; Golombok et al., 2014). Older children are more likely than younger children to experience greater difficulties settling into their new adoptive family and their adoptive parents correspondingly are likely to experience higher levels of parenting stress (Tornello et al., 2011; Goldberg and Smith, 2014). Further, gay fathers were found to have predominantly adopted boys, who in turn are generally more likely to display conduct problems than girls (Averett et al., 2009; Goldberg, 2009; Golombok et al., 2014). Transracial adoption and adoption of ethnic minority children also have been more prevalent among lesbian and gay parents when compared to heterosexual parents (Farr and Patterson, 2009; Lavner et al., 2012). Children in care from ethnic minorities were more likely than children in care from other ethnic backgrounds to have faced greater early adversity associated with more problem behaviors (e.g., Frazer and Selwyn, 2005).

The only longitudinal study of adoptive families in the United Kingdom (Golombok et al., 2014; McConnachie et al., 2021), thus far has focused exclusively on adopted children’s psychosocial adjustment and parent–child relationships, thereby excluding an examination of lesbian and gay parents’ perspectives on their assessment as prospective adopters. A recent United Kingdom survey about the motivations for adoption and experiences of 366 LGBT + adoptive parents and prospective adopters found that over two thirds of the sample did not expect to be discriminated or treated differently in the adoption assessment process (Costa and Tasker, 2018). However, close to 50% of the sample did expect to be matched with hard-to-place children, with lesbian women being more likely than gay men to report that they had indeed adopted hard-to-place children (Costa and Tasker, 2018). Similar findings were reported in an earlier study, in which some adoptive lesbian and gay parents reported experiencing prejudice within the United Kingdom adoption system (Mellish et al., 2013).

Taken together, this evidence raises the possibilities of lesbian and gay adopters being treated differently within the adoption system or being more likely to be placed with children who are considered hard-to-place, or perhaps a combination of both processes. Although openly discriminatory practices within the United Kingdom adoption system appear to have declined, implicit biases against LGBT + prospective adopters may persist as well as prospective parents’ internalized beliefs that they can only be placed with hard-to-place children.

### The Present Study

This study examined a developmental systems perspective in which expects that child development is influenced by parental characteristics and child’s characteristics in turn influence parental responses. These bidirectional relationships between children and their parents create unique family dynamics that determine the development of child problem behaviors and overall psychological adjustment (Cowan et al., 1996; Lamb, 2012). Further, a resilience-based perspective approaches family functioning as capable of adapting to difficulties and recovering from challenges, particularly in relation to adoption, with a focus on family strengths (Walsh, 2003, 2016). Within this theoretical perspective, we posit that both family processes in the adoptive family and the child’s preadoptive history affect child developmental outcomes rather than either family configuration or parental sexual identity. Given this evidence, the purpose of our study was twofold:

(1) To examine the characteristics of children placed with lesbian, gay, and heterosexual adopters, specifically whether lesbian and gay parents were more likely than heterosexual parents to have adopted hard-to-place children;
(2) To examine adopted child’s problem behaviors and positive psychosocial adjustment in lesbian, gay, and heterosexual parented families, while considering both the children’s preadoptive history and adoptive parenting behaviors across the three family types.

## Materials and Methods

### Participants

The initial sample was composed of 253 adoptive parents in the United Kingdom who had adopted at least one child through domestic adoption from public care. One parent per family completed an online survey [omitted for peer review] about the adoption process, their family dynamics, and their adopted child’s psychosocial adjustment. The initial sample comprised 176 were families headed by parents who identified as heterosexuals, 39 headed by fathers [37 gay, 1 bisexual, and 1 queer], 40 were headed by mothers [33 lesbian, 5 bisexual, 1 queer], and 1 family was headed by a parent who identified as transgender. In order to make comparisons across family types (heterosexual × lesbian × gay), three subsamples of adoptive families were formed, and matched using the propensity score method based on (a) Parent variables: type of adoption (single/couple), age, education level, economic level, and place of residence; (b) Child variables: age and gender, for a final sample of 149 adoptive parents (71 heterosexual parents, 39 lesbian mothers, and 39 gay fathers). Inclusion criteria for this study were that: (1) the respondent parent had adopted at least one child (target child); (2) the target child had been adopted at least 12 months prior to participation; and (3) the target child’s age at time of survey was between 5 and 18 years. In families with more than one adopted child, the selected *target child* was the oldest child who fulfilled the inclusion criteria.

As shown in Table 1, gay fathers were significantly younger, had a significantly higher annual income, and were more likely to be full-time employed than either heterosexual or lesbian parents. Heterosexual parents were significantly more likely to have adopted singularly than either lesbian or gay parents. Across family groups there were no differences in parental education, place of residence, total number of children in the family, or number of children adopted. The majority of parents adopted children as a couple and were married or in a civil partnership when they completed the survey. Further, the overwhelming majority of parents were White, with a college degree, and in full-time employment. There was an almost even distribution regarding place of residence, although around a third of families reported living in a town or small city. About a third of the parents had at least one non-adopted child, around two thirds had adopted one or two children, and the majority of parents (79%) had adopted younger children (between 5 and 12 years old).

**TABLE 1:** Sociodemographic data including parent and children’s main characteristics.

	**Heterosexual parents**		**Gay (bi and queer) fathers**		**Lesbian (bi and queer) mothers**		**Comparison tests**
	***n* = 71**		***n* = 39**		***n* = 39**		
	
**Parent variables**							
**Type of adoption**							*p*_A_ = 0.045; *p*_B_ = 0.031**
Single	16	23%	3	8%	6	15%	
Couple	55	77%	36	92%	33	85%	
Age (in years)*							***F*(2,148) = 7.031, *p* = 0.001**
Range, *M* (*SD*)	33–61	47.04 (6.59)	27–60	41.38 (7.53)	30–62	45.74 (9.35)	
**Relationship**							*p*_A_ = 0.164; *p*_B_ = 0.174**
Married	49	69%	15	39%	14	36%	
Civil partnership	0	0%	13	33%	11	28%	
Living together	5	7%	7	19%	4	10%	
Single/Separated	17	23%	4	9%	10	25%	
**Ethnicity**							*p*_A_ = 0.067; *p*_B_ = 0.098**
White British	63	89%	29	74%	33	85%	
White Other	7	10%	9	23%	2	5%	
Mixed White/Black	0	0%	0	0%	1	3%	
Mixed Other	0	0%	0	0%	1	3%	
Black/Black British	0	0%	0	0%	2	5%	
Asian/Asian British	1	1%	1	3%	0	0%	
**Education**							*p*_A_ = 0.433; *p*_B_ = 0.439**
GSCE	0	0%	2	5%	0	0%	
A/S or A level	3	4%	3	8%	0	0%	
Diploma or NVQ	5	7%	3	8%	5	13%	
BSc/BA	35	49%	13	33%	14	36%	
Masters	15	21%	13	33%	12	31%	
Doctorate	10	14%	4	10%	4	10%	
Other	3	4%	1	3%	3	8%	
**Employment**							*p*_A_ = 0.001; *p*_B_ = 0.001**
Full time	54	74%	32	82%	28	72%	
Unemployed/Retired	3	6%	2	5%	5	13%	
Stay-at-home	7	10%	2	5%	2	5%	
Other	7	10%	3	8%	4	10%	
Income*							***Kw*(2) = 14.314, *p* = 0.001**
£0–£32,000	17	24%	1	2%	14	36%	
£32,001–£150,000	50	70%	35	90%	25	64%	
> £150,000	4	6%	3	8%	0	0%	
**Place of residence**							*p*_A_ = 0.037; *p*_B_ = 0.039**
Large city	8	11%	14	36%	11	28%	
Suburbs large city	12	17%	5	13%	8	21%	
Town/Small city	32	45%	14	36%	12	31%	
Country village	19	27%	6	15%	8	21%	
**Non-adopted children**							*F*(2,148) = 0.846, *p* = 0.431
0	48	67%	30	77%	25	64%	
1	15	21%	4	10%	6	15%	
2	4	6%	4	10%	6	15%	
3	4	6%	1	3%	1	3%	
4	0	0%	0	0%	1	3%	
**Adopted children**							*F*(2,148) = 0.914, *p* = 0.403
1	42	59%	20	47%	18	46%	
2	24	34%	14	14%	19	49%	
3	4	6%	4	11%	2	5%	
4	1	1%	0	0%	0	0%	
5	0	0%	0	0%	0	0%	
6	0	0%	1	3%	0	0%	

*Significant differences between groups; Statistics in bold highlight significant differences. GCSE, General Certificate of Secondary Education; NVQ, National Vocational Qualification; BSc, Bachelor of Sciences; BA, Bachelor of Arts. **Fisher’s Exact Test for 2 × 3 contingency tables are in fact Fisher–Freeman–Halton Exact Test (Freeman and Halton, 1951).

### Measures

Participants completed the [omitted for peer review] online survey comprising detailed questionnaires on the adoption process, family sociodemographic information, and the target child’s preadoptive history. Further information about parenting practices and child’s adjustment was collected through standardized psychological measures.

#### Hard-to-Place Children

Hard-to-place children’s characteristics were assessed through the adoptive parent’s assessment of the following variables: (1) *Prenatal adversity* (birth mother’s prenatal use of drugs/alcohol – “yes/no” format); (2) *Pre-care experiences* [(a) physical neglect, (b) physical abuse, (c) sexual abuse, (d) emotional neglect – “yes/no” format]; (3) *Care experiences* [(a) age when taken into care, (b) age at adoption, (c) number of institutional placements, (d) number of foster placements, (e) duration of placements – in months]; (4) *Preadoption problems* [(a) physical problem/disability, (b) learning disability, (c) psychological problem – “yes/no” format]; (5) *Gender* (boy/girl/other); (6) *Ethnicity* (child from an ethnic minority – United Kingdom census ethnic categories); (7) *Sibling group adoption* (child adopted as part of a sibling group – “yes/no” format). Although these data were indirectly collected through the respondent parent, United Kingdom law mandates that when information is known, adoption workers share all the above information with the adoptive parents. Prenatal adversity, pre-care experiences, preadoption problems, ethnicity, sibling group adoption, and gender were coded as dummy variables so that 0 = no and 1 = yes, and gender as 0 = girl and 1 = boy. Care experiences were measured as continuous variables. Two final questions about the times for approval as prospective parents and for child placement were posed; (1) “How long did you wait from the moment you applied to be an adoptive parent until you were approved to adopt,” and (2) “How long did you wait from the moment you were approved as an adoptive parent until the time your child came to live with you,” both measured in months.

#### Parenting Behaviors

The Parenting Behaviors Inventory (Patterson et al., 1992; Costa et al., 2012) was used to measure the current parenting practices. Two positive dimensions of parenting behaviors were used in this study: Closeness (e.g., item 3 “In the evening I talk with my children about the past and the coming day”; α = 0.855), and Rules (e.g., item 13 “I teach my children to obey rules”; α = 0.848). Items assess how often each parenting behavior occurred and are measured on a 5-point Likert scale that ranged from 1 (*never*) to 5 (*always*). Mean scores were computed so that higher scores on each scale reflect higher frequencies of closeness and rule setting behaviors.

#### Children’s Psychosocial Adjustment

The Strengths and Difficulties Questionnaire (SDQ; Goodman, 1997, 1999) was used to measure child’s behavioral or emotional problems and prosocial behaviors. For this study, the three-factor model consisting of Internalizing Problems (e.g., item 3 “Often complains of headaches, stomach-aches or sickness”; α = 0.832), Externalizing Problems (e.g., item 2 “Restless, overactive, cannot stay still for long”; α = 0.835), and Prosocial Behaviors (e.g., item 1 “Considerate of other people’s feelings”; α = 0.829) was used, as recommended for low-risk community samples (Goodman et al., 2010). Items assess to what extent each situation characterized the child during the previous 6 months and were measured on a 3-point Likert scale that ranged from 0 (*not true*) to 2 (*certainly true*). Mean scores were computed so that higher scores on the Internalizing and Externalizing dimensions reflect higher levels of problem behaviors whilst a higher score on the prosocial dimension reflects greater social competence.

The Positive and Negative Affect Schedule (PANAS; Watson and Clark, 1994) was used to measure child’s emotional adjustment, consisting of two broad dimensions of Positive Affect (e.g., item 3 “Attentive”; α = 0.863) and Negative Affect (e.g., item 7 “Irritable”; α = 0.925). Items assess the extent to which each emotion described the child during the previous weeks and were measured on a 5-point Likert scale that ranged from 1 (*very slightly or not at all*) to 5 (*extremely*). Mean scores were computed so that higher scores on the Positive Affect dimension reflect higher frequency of positive emotions whilst higher scores on the Negative Affect dimension reflect higher frequency of negative emotions.

### Procedures

This study was part of a nationwide research project concerning the psychosocial development and well-being of adopted children. The [omitted for peer review] survey was advertised through adoption agencies and local authorities who had placed children for adoption across England, Scotland, Wales, and Northern Ireland. To boost cell sizes, LGBT + support groups and LGBT + parent groups were also contacted and asked to send out information about the study to their members. Therefore, it was not possible to obtain information on the participation rate. Consent forms were collected from all participants prior to completing the survey.

The research protocol was developed in close collaboration with a team of experts, namely stakeholders from adoption and fostering agencies, a national LGBT + adoptive parents group, a private consultant in the field of adoption, and two independent adoptive parents. The experts were briefed on the design and the aims of the study and were asked for their feedback on the items included in the survey assessing the adoption process and the child’s preadoption history. The [omitted for peer review] study was approved by the [omitted for peer review] Ethics Committee.

### Analysis Plan

All statistical analyses were performed using the software IBM SPSS Statistics (v. 25, IBM Corp., Armonk, NY, United States). Before proceeding with the analyses, that data was examined for missing values. No changes were made to few missing values on parents’ characteristics, children’s characteristics, or adoption data. Missing values found on responses on standardized scales (Parenting Behaviors Inventory, Strengths and Difficulties Questionnaire, and Positive and Negative Affect Schedule) were imputed when its frequency was lower than 10%. This was done using the mean interpolation method.

To address the first objective of this study, the first set of analyses compared children’s hard-to-place characteristics among the three family groups through contingency tables using Fisher’s exact tests and one-way ANOVAs. In addition, to compare parents’ preferences and specified adoption criteria among the three family groups, Fisher’s exact tests were conducted. To address the second objective of this study, General Linear Models (GLM) were conducted to compare parenting behaviors and children’s psychosocial adjustment in the three family groups, and to examine the effects of family type and parenting behaviors on children’s psychosocial adjustment.

## Results

### Hard-to-Place Children and Adoption Placement Practices

#### Hard-to-Place Children

The three family groups were compared regarding children’s hard-to-place characteristics (Table 2). No differences were found across the family groups regarding children’s prenatal adversity experiences (birth mother’s use of drugs/alcohol) and pre-care experiences (e.g., neglect). Regarding adopted children’s care experiences, no differences among family groups were found on number of institutional placements or duration of previous placements. However, compared to lesbian or heterosexual parents, gay fathers were significantly more likely to have adopted children who were older when taken into care, and also those who were older at time of adoption. In contrast, heterosexual parents were more likely to have adopted children who had fewer foster care placements than those adopted by either lesbian or gay parents. Regarding children’s pre-adoption characteristics, no significant differences were found across the family groups – except for gay fathers, who were less likely than the other parents to have adopted children with a physical disability. Heterosexual, gay, and lesbian parents were all just as likely to have adopted a sibling group. Nevertheless, gay fathers were more likely to have adopted boys than either lesbian or heterosexual parents, whereas lesbian mothers were more likely to have adopted a child from an ethnic minority then were either gay or heterosexual parents.

**TABLE 2:** Comparisons across family groups on children’s hard-to-place characteristics.

	**Heterosexual parents *n* = 71**	**Gay (bi and queer) fathers *n* = 39**	**Lesbian (bi and queer) mothers *n* = 39**	**Comparison tests**
(1) Prenatal adversity	25 (49%)	17 (61%)	12 (44%)	*p*_A_ = 0.449; *p*_B_ = 0.441**
(2) Pre-care experiences				
(a) Physical neglect	49 (69%)	29 (81%)	28 (72%)	*p*_A_ = 0.445; *p*_B_ = 0.470**
(b) Physical abuse	20 (28%)	13 (36%)	12 (31%)	*p*_A_ = 0.702; *p*_B_ = 0.716**
(c) Sexual abuse	6 (9%)	1 (3%)	5 (13%)	*p*_A_ = 0.285; *p*_B_ = 0.283**
(d) Emotional neglect	50 (71%)	29 (81%)	27 (69%)	*p*_A_ = 0.464; *p*_B_ = 0.498**
(3) Care experiences				
(a) Age taken into care	23.44 (22.40)	35.65 (27.00)*	18.22 (18.45)	***F*(2,131) = 5.27, *p* = 0.006**
(b) Age at adoption	39.70 (25.99)	58.58 (26.45)*	39.92 (19.83)	***F*(2,131) = 6.95, *p* = 0.001**
(c) Number inst. placements	0.46 (0.80)	0.16 (0.46)	0.38 (0.76)	*F*(2,131) = 1.79, *p* = 0.170
(d) Number foster placements	1.48 (0.88)*	1.87 (1.18)	2.03 (1.19)	***F*(2,131) = 3.62, *p* = 0.029**
(e) Duration of placement	16.24 (7.69)	18.35 (11.11)	18.76 (10.73)	*F*(2,131) = 1.01, *p* = 0.368
(4) Pre-adoption problems				
(a) Physical problem	22 (31%)	3 (8%)*	10 (26%)	*p*_A_ = 0.033; *p*_B_ = 0.003**
(b) Learning disability	21 (30%)	11 (31%)	16 (41%)	*p*_A_ = 0.447; *p*_B_ = 0.456**
(c) Psychological problem	32 (46%)	11 (31%)	20 (51%)	*p*_A_ = 0.169; *p*_B_ = 0.179**
(5) Gender (boy)	41 (58%)	32 (89%)*	19 (49%)	*p*_A_ < *0.001*; *p*_B_ < 0.001**
(6) Ethnicity (minority)	8 (11%)	5 (14%)	13 (33%)*	*p*_A_ = 0.010; *p*_B_ = 0.010**
(7) Sibling group (yes)	22 (31%)	16 (44%)	16 (41%)	*p*_A_ = 0.663; *p*_B_ = 0.666**

*Significant differences between groups; Statistics in bold highlight significant differences. **Fisher’s Exact Test for 2 × 3 contingency tables are in fact Fisher–Freeman–Halton Exact Test (Freeman and Halton, 1951).

#### Adoption Placement Practices

Asked if they had any criteria regarding children’s characteristics, 105 (71%) parents stated they had specified one of more criteria regarding the children they wanted to adopt. No significant differences between the three family groups regarding the number of criteria they had specified were found (Table 3). For the whole sample, age was the most prevalent criterion expressed in that 91 (87%) wanted to adopt a child within a specific age group. Further, 20 parents (19%) wanted to adopt a child with no physical or psychological disability, 14 (13%) wanted a child from a specific ethnic background, and 3 (3%) stated that they did not want a child who had suffered either prenatal or early adversity. When comparing the three family groups, significant differences were found for only two criteria: Heterosexual parents (78%) were more likely than either lesbian or gay parents to specify they did not want to adopt a child with any hard-to-place characteristic. Further, gay fathers were more likely than heterosexual and lesbian parents to have specified wanting to adopt a boy. In fact, of the gay fathers who had specified a gender preference (21%), all specified wanting to adopt a boy. No differences between the groups were found on child’s age preference, willingness to adopt a sibling group, or unwillingness to adopt children with a disability, or a child who had faced pre-care adversity. Of note, no significant between-group differences were found for ethnic preference, although some lesbian mothers and gay fathers had specified that they were open to adopt a child from an ethnic minority background. In contrast, of the few heterosexual parents who had expressed an ethnicity preference, all had specified wanting to adopt a White child. Inspection of the individual data revealed that all but one lesbian mother and all but one gay father who had been open to adopting a child from an ethnic minority background themselves belonged to an ethnic minority group or were a partner in a mixed-ethnicity couple. Lastly, the months until approved as a prospective adopter were compared with no differences found between family groups, *F*(2,146) = 0.366, *p* = 0.684 (*M* = 17.65, *SD* = 11.63). However, differences were found regarding the waiting time from approval until child placement, *F*(2,143) = 4.502, *p* = 0.013. Heterosexual parents waited significantly longer (*M* = 12.77) than did lesbian mothers (*M* = 6.69) to be matched with a child; However, no significant differences were found between gay fathers (*M* = 9.31) and the other two family groups.

**TABLE 3:** Parents’ specified adoption criteria.

	**Heterosexual parents *n* = 71**	**Gay (bi and queer) fathers *n* = 39**	**Lesbian (bi and queer) mothers *n* = 39**	**Fisher-Freeman-Halton Exact Test**
Specified 1 or more criteria	50 (71%)	26 (67%)	28 (72%)	*p*_A_ = 0.874; *p*_B_ = 0.920
No hard-to-place criteria	55 (78%)*	21 (54%)	22 (56%)	***p*_A_ = 0.016; *p*_B_ = 0.018**
Gender	20 (28%)	8 (21%)*	6 (15%)	
(a) Boy	11 (55%)	8 (100%)	1 (17%)	***p*_A_ = 0.013; *p*_B_ = 0.011**
(b) Girl	9 (45%)	0	5 (83%)	
Age	43 (61%)	24 (62%)	23 (59%)	
(a) Younger (up to 4yo)	32 (74%)	14 (58%)	19 (83%)	*p*_A_ = 0.161; *p*_B_ = 0.188
(b) Older (over 4yo)	11 (26%)	10 (42%)	4 (17%)	
Ethnicity	3 (4%)	4 (10%)	7 (18%)	
(a) White	100%	1 (20%)	2 (29%)	*p*_A_ = 0.117; *p*_B_ = 0.178
(b) Minority	0	4 (80%)	5 (71%)	
Sibling group	6 (9%)	5 (13%)	6 (15%)	*p*_A_ = 0.522; *p*_B_ = 0.501
No disability	9 (13%)	3 (8%)	8 (21%)	*p*_A_ = 0.244; *p*_B_ = 0.284
No pre-care adversity	2 (3%)	0	1 (3%)	*p*_A_ = 0.579; *p*_B_ = 0.800

Statistics in bold show significant differences. *Highlights group that is significantly different.

### Parenting Behaviors and Children’s Psychosocial Adjustment

General Linear Models were conducted to compare parenting behaviors and children’s psychosocial adjustment in lesbian, gay, and heterosexual parents. In the first GLM, the three adoptive family groups were compared on parenting behaviors and no significant differences were found for either parental closeness, *F*(2,149) = 0.600, *p* = 0.550, η^2^_p_ = 0.008, or parental rule setting, *F*(2,149) = 1.268, *p* = 0.284, η^2^_p_ = 0.017. In the second GLM, children’s problem behaviors (Externalizing, Internalizing, and Negative Affect) were introduced as outcomes, and likewise in the third GLM, children’s positive adjustment (Prosocial Behaviors and Positive Affect) were introduced as outcomes (Table 4). No significant differences between the three family groups on children’s problems or positive adjustment were found; specifically, for Externalizing, *F*(2,149) = 0.649, *p* = 0.524, η^2^_p_ = 0.009, Internalizing, *F*(2,149) = 0.257, *p* = 0.774, η^2^_p_ = 0.004, Negative Affect, *F*(2,149) = 0.198, *p* = 0.821, η^2^_p_ = 0.003, Prosocial Behaviors, *F*(2,149) = 0.681, *p* = 0.508, η^2^_p_ = 0.009, or Positive Affect, *F*(2,149) = 0.050, *p* = 0.951, η^2^_p_ = 0.001.

**TABLE 4:** Comparisons across family groups on parenting behaviors and children’s psychosocial adjustment.

	**Heterosexual parents**	**Gay (bi and queer) fathers**	**Lesbian (bi and queer) mothers**
Parental closeness	4.34 (0.51)	4.35 (0.47)	4.44 (0.40)
Parental rules	4.55 (0.55)	4.58 (0.37)	4.42 (0.45)
Externalizing problems	2.06 (0.47)	1.96 (0.48)	2.05 (0.47)
Internalizing problems	1.77 (0.49)	1.72 (0.46)	1.81 (0.56)
Negative affect	2.50 (1.04)	2.41 (1.01)	2.55 (0.90)
Prosocial behaviors	2.24 (0.53)	2.18 (0.53)	2.32 (0.57)
Positive affect	3.36 (0.78)	3.39 (0.83)	3.33 (0.67)

To examine the effects of family type and parenting behaviors on child problem behaviors and positive psychosocial adjustment after controlling for children’s preadoptive history, a series of hierarchical multiple regression analyses were conducted. Family group was introduced in step one. Step two introduced children’s preadoptive variables that significantly differed across the three family groups (age when taken into care, number of foster care placements, physical problem, gender, and ethnic background; Table 4). Parental closeness and parental rule setting were introduced in the final step. The outcome variables were children’s scores on externalizing behaviors, internalizing behaviors, negative affect, prosocial behaviors, and positive affect (Table 5).

**TABLE 5:** Analyses of predictors of children’s psychosocial adjustment.

	**Externalizing behaviors**			**Internalizing behaviors**			**Negative affect**			**Prosocial behaviors**			**Positive affect**		
	***R*^2^_Adj_**	***B***	**β**	***R*^2^_Adj_**	***B***	**β**	***R*^2^_Adj_**	***B***	**β**	***R*^2^_Adj_**	***B***	**β**	***R*^2^_Adj_**	***B***	**β**
Step one	–0.01			–0.01			–0.01			–0.01			–0.01		
Family type: Gay vs. heterosexual		–0.11	–0.10		–0.06	–0.05		–0.11	–0.05		–0.06	–0.05		0.01	0.01
Family type: Lesbian vs. heterosexual		–0.02	–0.02		0.03	0.03		0.03	0.02		0.08	0.06		–0.04	–0.03
Step two	0.07			0.11			0.07			0.01			–0.03		
Age taken care		–0.01	–0.01		**–0.01**	–0.16*		0.01	0.14		–0.01	–0.01		–0.01	–0.15
N foster placements		**0.09**	0.20*		**0.13**	0.26*		**0.19**	0.20*		–0.08	–0.15		–0.01	–0.01
Physical disability		**0.30**	0.27*		**0.35**	0.30*		**0.59**	0.25*		**–0.22**	–0.17*		–0.15	–0.08
Gender		0.02	0.02		0.01	0.01		–0.02	–0.01		–0.04	–0.04		–0.01	–0.01
Ethnic minority		–0.06	–0.05		–0.08	–0.06		0.36	0.14		–0.02	–0.01		–0.03	–0.02
Step three	0.06			0.11			0.07			0.03			0.06		
Parental closeness		–0.09	–0.09		–0.06	–0.06		–0.22	–0.10		**0.23**	0.20*		**0.47**	0.29*
Parental rules		–0.01	–0.01		–0.04	–0.04		–0.01	–0.01		–0.03	–0.03		0.09	0.06

*Highlights significance. Statistics in bold highlight significancy.

Number of foster care placements (*p* = 0.019; *p* = 0.001; *p* = 0.017) and physical problem/disability (*p* = 0.002; *p* < 0.001; *p* = 0.003) were found to predict children’s externalizing and internalizing behaviors and negative affect, respectively, with a greater number of preadoption foster care placements and the presence of physical problems/disability being associated with more problem behaviors and negative affect. Additionally, age when taken into care was found to predict children’s internalizing problems (*p* = 0.050), with older age when taken into care being associated with greater internalizing problems. None of the parental characteristics emerged as significant predictors of internalizing or externalizing behaviors or of negative affect. In terms of positive adjustment, not having a physical disability (*p* = 0.050) significantly predicted prosocial behaviors in that the absence of a physical problem was associated with more prosocial behaviors. Parental closeness (*p* = 0.040; *p* = 0.002) significantly predicted both prosocial behaviors and positive affect, respectively, in that greater warmth in parent-child relationships was associated with more prosocial behaviors and greater positive affect.

## Discussion

This study had two main objectives. The first objective was to examine if lesbian and gay adopters have disproportionally adopted hard-to-place children when compared to heterosexual adopters, and if so, if that could be explained by parents’ preferences or possibly due to differential and discriminatory adoption processes. The second objective was to examine adopted children’s problem behaviors and positive psychosocial adjustment across the three family types and investigate both preadoption adversity and adoptive parenting variables that may explain the relationships between adoption placement and children’s outcomes. Further, an innovative approach in this study controlled for differences in prenatal adversity across family groups that previous studies have not. While some evidence was found that lesbian and gay parents were more likely than heterosexual parents to adopt hard-to-place children, no differences in children’s outcomes were found across the three family types. This finding is particularly noteworthy given the greater likelihood of lesbian and gay parents adopting children who had faced greater adversity during their early lives, and therefore may have displayed greater adjustment difficulties.

Early research in the United States and in the United Kingdom has found some evidence of differential treatment of lesbian/gay and heterosexual parents within the adoption system insofar as lesbian/gay parents faced greater scrutiny during the adoption assessment process, experienced prejudice, and seemed more likely to adopt children who had faced greater preadoption adversity and may have had greater physical, emotional, or behavioral difficulties as a consequence (e.g., Hicks, 1996; Brooks and Goldberg, 2001). However, these studies were conducted over 10 years ago, and important societal and legal advances have been made since then. In the present study it was found that gay fathers were more likely to have adopted boys, older children, and children who were older when taken into care and experienced more foster care placements. We were able to examine why this might have happened by contrasting children’s placements with the criteria that gay fathers had specified upon application. It was noticeable that gay fathers were more willing to adopt boys, and generally more open than either lesbian or heterosexual parents to adopting older children. For lesbian mothers, who were more likely than either heterosexual or gay parents to have adopted a child from an ethnic minority group, again this difference seems to reflect stated preferences and openness to adopt ethnic minority children. In general, birth parents favor having boys rather than girls, and this gender preference has been observed in different cultures (e.g., in Europe, North Africa; Brockmann, 2001; Rossi and Rouanet, 2015). In contrast, adoptive parents tend to prefer adopting girls rather than boys, with the exception of gay men (Goldberg, 2009; Golombok et al., 2014). This apparent paradox might be explained by two contrasting factors; One the one hand, a preference for having boys may be a consequence of highly gendered societies, which tend to value men and masculinity traits. One the other hand, prospective adopters - heterosexuals in particular – may be more aware that boys are more likely to display adjustment and behavior problems which makes them less desirable as adoptive children. The exception of gay adoptive fathers seems to be related to a greater self-efficacy and confidence in socializing a child of their own gender (Goldberg, 2009).

Nevertheless, the fact that lesbian and gay parents have adopted children with hard-to-place profiles did not appear to support the argument of a preferential treatment of heterosexual parents within the adoption system, but instead appeared to indicate an adoptive matching process that reflected lesbian and gay parents’ openness and willingness to adopt children who adoption services usually found were hard-to-place. However, it should be noted that some lesbian and gay adopters may have stated a greater openness to adopt children with hard-to-place profiles because they may have internalized that they would not otherwise be placed with a child after years of being denied this possibility or overly scrutinized regarding their parenting abilities. As previous research has shown, lesbian and gay prospective adopters were for a long time perceived as a ‘last resort’ for children who would not be adopted by heterosexual people (Hicks, 1996).

In addition, we found that heterosexual parents tended to wait longer than gay fathers, and significantly longer than lesbian mothers, to be placed with a child. The significant of this finding is difficult to explain within this study, but we speculate that the stated preferences and/or openness of lesbian and gay parents to adopt children who are regarded as being more difficult to place could facilitate the matching process between parents and child and accelerate placement times. Other indirect evidence of differential placement practices was not found in that lesbian, gay, and heterosexual parents waited a similar amount of time to be approved as prospective adopters. As we are reliant on retrospective self-report data, a direct assessment of adoption agencies’ practices, in particular of the factors that are taken into account in matching children with prospective adopters, may further illuminate these tentative findings.

The hard-to-place profiles of some of the adopted children were associated with children’s negative but not positive outcomes across all family types. Specifically, number of foster care placements and physical disability were associated with all three negative emotional and behavioral outcomes, with level of internalizing problems additionally being associated with age when taken into care. Nevertheless, no differences were found between lesbian, gay, and heterosexual adopters regarding their children’s internalizing or externalizing problems, negative affect, prosocial behaviors or positive affect. Here, the absence of differences in children’s outcomes as a function of parental sexual orientation is in line with studies from the United States among younger children under 6 years old (e.g., Farr et al., 2010, *M*_age_ = 3; Goldberg and Smith, 2014, *M*_age_ = 2.5). A previous United Kingdom study (Golombok et al., 2014) with children aged between 3 and 9 years, reported that those adopted by heterosexual parents displayed greater externalizing problems than those adopted by lesbian/gay parents, although a follow-up study of the children in Golombok’s study when aged between 10 and 14, failed to identify any differences in their psychosocial adjustment between the three family types (McConnachie et al., 2021).

Regarding the association of children’s psychosocial adjustment with parenting behaviors, two distinctive patterns of findings were identified in this study. First, we found no association of parental sexual orientation with children’s negative outcomes (internalizing problems, externalizing problems, or negative affect). It was found that the disadvantages created by children’s preadoption experiences were associated with negative behavioral and emotional adjustment. Prenatal adversity, negligence and abuse in the biological family, and disruption in preadoption placements were all linked to problem behaviors and negative affect, in accordance with previous research (Rutter, 2000; Palacios and Brodzinsky, 2010). Further, children’s difficulties, namely, learning disabilities and psychological problems were significantly associated with greater internalizing and externalizing problems, negative affect, and lower levels of prosocial competences.

A second pattern of findings showed that good adoptive parenting may have boosted children’s positive adjustment, in particular parental closeness (i.e., being involved in children’s activities and making time to listen to them) was associated with higher scores of children’s prosocial behavior and positive affect. Whereas previous research mostly highlighted the effects of parental stress on adopted children’s problem behaviors (e.g., Goldberg and Smith, 2014; Golombok et al., 2014), or the effects of the quality of coparenting and couple relationship on the absence of children’s problem behaviors (e.g., Farr et al., 2010, 2020), this study examined positive parenting practices (parental closeness and parental rule setting) and the association of these with both children’s problem behaviors and positive adjustment.

Research on positive adjustment and resilience of adopted children is still in its early stages (Palacios and Brodzinsky, 2010; Grotevant and McDermott, 2014). We suggest that future adoption research should consider the positive processes associated with successful adoptions to help to illuminate a clearer picture including positive child adjustment. Given the differences found in the placement of children with lesbian, gay, and heterosexual adopters in face of the absence of difference in child outcomes, it is plausible to suggest that lesbian and gay parents may make use of unique competences to promote their adopted children’s psychosocial adjustment. Lesbian and gay prospective adopters may be more willing and better prepared to face the demands of raising children with a difficult past by fostering a positive adjustment after significant adversity (Walsh, 2003). Further, as Ausbrooks and Russell (2011) argued, gay and lesbian adoptive parents may be more equipped to parent an ethnic minority child by drawing from their own experiences of being a minority insofar as the challenges associated with being a sexual minority may promote lesbian and gay people’s (and their children’s) resilience in face of stress, stigma, and adversity (Meyer, 2015).

### Limitations and Strengths

The present study had several limitations that must be acknowledged. First, this study utilized a non-random intentional sample, which limits the generalizability of its findings to other adoptive families. A second limitation regards the cross-sectional design of this study, thus causality cannot be ascertained and our findings can only denote associations. Longitudinal studies of adoptive families including both parents and children are needed to investigate parental processes that may be specific to some family types and the effects of these upon children’s developmental outcomes. Some longitudinal studies on adoption by lesbian, gay, and heterosexual parents have started to emerge in the United States (e.g., Goldberg and Smith, 2013; Farr, 2017), and one in the United Kingdom (e.g., Golombok et al., 2014; McConnachie et al., 2021), and these are expected to shed some light onto the similar and unique family processes across different family configurations. Further, parent-child relationships are bidirectional, thus child characteristics should also be taken into account as potentially affecting parental responses and behaviors. A third limitation pertains to the fact that only one parent per household completed the survey, thus providing data for all the variables measured. The reliance on parental report alongside the online data collection adopted in this study calls for caution when considering these findings. Nevertheless, these findings are aligned with those from both the United States and the United Kingdom. Data from multiple informants including the child, the child’s other adoptive parent (when applicable), alongside independent data from teachers or researcher observational data could further elucidate family processes associated with child adjustment. Lastly, the age range of the children included in this study was wide. Although the measures of psychosocial adjustment used were age appropriate, these may lack precision for children at different developmental stages. The modest number of gay and lesbian parented families prevented a closer examination of possible age cohort similarities and differences.

Despite these limitations, this study had also important strengths. First, the sample was diverse regarding both parental characteristics (e.g., single and couple adoption, geographical distribution) and children’s characteristics (e.g., age range, preadoption experiences), thus adding a wider perspective to the literature on adoption and family processes. Although this diversity may add unwanted variability that could not be fully controlled, it does suggest developmental trajectories and family processes after the adoption placement that should be further examined as older children may be doing as well as younger children in their adoptive families. Most studies comparing lesbian, gay, and heterosexual adopters have focused on young children who may not have faced as many preadoption difficulties as those adopted at latter ages (e.g., Golombok et al., 2014). Moreover, older children are likely to have a clearer understanding of their identity, and this may affect both family dynamics and their psychological adjustment. By including both younger and older adopted children, this study extended research on the psychosocial adjustment of children adopted by lesbian and gay parents. Lastly, the innovatory focus of the study on both positive and negative child outcomes and associations with adoptive parenting processes illuminated adaptive and resilient processes displayed across lesbian, gay, and heterosexual adoptive parent families to indicate why adopted children show similar gains in positive socioemotional development despite any initial disadvantage associated with their hard-to-place profiles. Future research should focus on examining not only the differences between gay, lesbian, and heterosexual parents and their adopted children, but also the specificities of gay and lesbian parented families and the family processes that enable hard-to-place children to overcome the negative effects of early adversity.

## Data Availability Statement

The raw data supporting the conclusions of this article will be made available by the authors, without undue reservation.

## Ethics Statement

The studies involving human participants were reviewed and approved by Comissão de Ética do ISPA – Instituto Universitário. This study was also approved by Birkbeck’s Ethics Committee. The patients/participants provided their written informed consent to participate in this study.

## Author Contributions

PAC, FT, and IPL: conception of the work, drafting the manuscript, final approval of the version to be published, and agreement to be accountable for all aspects of the work. PAC: acquisition of data and data analysis. PAC and FT: interpretation of data. All authors: contributed to the article and approved the submitted version.

## Conflict of Interest

The authors declare that the research was conducted in the absence of any commercial or financial relationships that could be construed as a potential conflict of interest.
